# Exercise may improve lung immunity after surgical stress: Evidence from a nephrectomy model via a bioinformatic analysis

**DOI:** 10.1371/journal.pone.0303334

**Published:** 2024-06-07

**Authors:** Min-You Wu, Hao-Lun Luo, Ya-Chuan Chang, Chia-Ying Yu, Wen-Wei Sung

**Affiliations:** 1 School of Medicine, Chung Shan Medical University, Taichung, Taiwan; 2 Department of Urology, Chung Shan Medical University Hospital, Taichung, Taiwan; 3 Department of Urology, Kaohsiung Chang Gung Memorial Hospital and Chang Gung University College of Medicine, Kaohsiung, Taiwan; 4 Center for Shockwave Medicine and Tissue Engineering, Kaohsiung Chang Gung Memorial Hospital and Chang Gung University College of Medicine, Kaohsiung, Taiwan; 5 Institute of Medicine, Chung Shan Medical University, Taichung, Taiwan; University of Vermont, UNITED STATES

## Abstract

Exercise offers numerous benefits to cancer patients and plays an essential role in postsurgical cancer rehabilitation. However, there is a lack of research examining the effects of exercise after the surgical stress of nephrectomy. To address this gap, we created an animal model that simulated patients who had undergone nephrectomy with or without an exercise intervention. Next, we performed a bioinformatic analysis based on the data generated by the RNA sequencing of the lung tissue sample. An overrepresentation analysis was conducted using two genome databases (Gene Ontology and Kyoto Encyclopedia of Genes and Genomes [KEGG]). A KEGG analysis of the exercise-treated nephrectomy mice revealed enrichment in immune-related pathways, particularly in the NF-κB and B cell-related pathways. The expression of CD79A and IGHD, which are responsible for B cell differentiation and proliferation, was upregulated in the nephrectomy mice. Differential gene expression was categorized as significantly upregulated or downregulated according to nephrectomy and exercise groups. Notably, we identified several gene expression reversals in the nephrectomy groups with exercise that were not found in the nephrectomy without exercise or control groups. Our preliminary results potentially reveal a genetic landscape for the underlying mechanisms of the effects of exercise on our nephrectomy model.

## Introduction

Exercise is widely recognized as a positive factor in cancer prevention, immune environment modulation, cognitive function improvement, and psychological health enhancement [1–4]. Evidence from previous epidemiological studies has revealed that exercise reduces the risk of several cancers and disease recurrence, especially in breast, prostate, and colon cancers [5–7]. Moreover, exercise enhances immunotherapy through immune cell production and tumor microenvironment regulation [8]. Furthermore, the adverse events of other cancer therapies are reduced by exercise interventions [4].

Anticancer agents and surgical resection cause several side effects in patients; therefore, cancer rehabilitation is essential for relieving side effects and improving the quality of life of post-treatment cancer patients [9]. Cancer rehabilitation, including physical exercise, emotional support, and music therapy, leads to better outcomes for cancer survival and physical activity [9, 10]. Physical exercise not only improves soft tissue impairment, such as lymphedema due to lymph node dissection, but also improves quality of life by reducing physical impairment [11]. Exercise training after cancer surgery promotes energy metabolism and reduces postoperative stress in mice with hepatocellular carcinoma [12]. However, to date, the effect of exercise in cancer patients after surgery has rarely been studied, and research has been limited to specific cancer types. Hence, further studies are warranted to uncover the effects of exercise training on different types of cancer to revise clinical approaches after surgery.

Renal cell carcinoma (RCC), which accounts for 90% of kidney cancers, consists of a set of cancers with different histological characteristics derived from the renal epithelium and is an incident cancer worldwide [13, 14]. The choice of therapy for RCC depends on the stage of the cancer. Patients with localized RCC generally undergo partial or radical nephrectomy or ablation intervention, while patients with metastatic RCC require systemic agent treatments [15]. To our knowledge, no study has yet attempted to discover the underlying mechanisms of the effects of exercise on a post-nephrectomy model by using a bioinformatics-based method. Our study targeted the investigation of the genetic impact of different exercise intensities on a post-nephrectomy animal model. Here, we first created an animal model that mimics a patient who has received a radical nephrectomy and then performed a bioinformatic analysis to explore the genetic influence of exercise in this animal model.

## Methods

### Post-nephrectomy animal model

The animal use protocol received approval from the Chung-Shan Medical University Experimental Animal Center (Protocol No. 2115), and all experiments were conducted in accordance with applicable guidelines and regulations. We selected 8-week-old male mice with a C57BL/6 genetic background to establish our animal model. On day 0, the mice underwent unilateral radical nephrectomy and were then randomly assigned to one of the following three groups: a no exercise group (Nx-con, N = 3), 30-minute exercise group (Nx-low, N = 4), and 60-minute exercise group (Nx-high, N = 4). Mice that underwent pseudo-surgery without exercise were assigned to a control group (Con) (S1 Fig).

### Treadmill exercise and training

Exercise training was performed with a treadmill exerciser for mice (T510E-C, ZGene Biotech Inc., Taipei, Taiwan) at a speed from 7 to 16 m per minute and with a 0° angle of inclination for 30 or 60 minutes. To reduce the stress impact on the mice, we set an adaption period before the experiment started (5 days for 10 minutes per day) using a speed of 5 m per minute. Furthermore, we pushed the mice slightly to urge them to exercise rather than using an electric stimulator. After the adaption period, nephrectomy was performed followed by a 3-day recovery period, and treadmill exercise was subsequently performed for 30 or 60 minutes per day for 5 consecutive days. The mice were sacrificed with CO_2_ after exercising on the fifth day of the experiment.

### Unilateral radical nephrectomy

The mice received Zoletil 50 (30 mg/kg) i.p. and xylazine (6 mg/kg) i.p. for analgesia. We checked the toe reflex to determine the depth of the anesthesia. After complete analgesia, we removed hair from the left flanks to the midline of the abdomen using an electric clipper and depilatory cream and used iodine to disinfect the surgical site. The mice were placed in a lateral position, and an incision was made 1 cm lateral to the last rib. The kidney was gripped, and the supplied vessels were ligated with a 4–0 nylon suture. After kidney resection, we confirmed that there was no bleeding at the site, and then the incision was sutured with a 4–0 nylon suture. The duration of the surgery was about 20 minutes. We injected cefazolin (50 mg/kg) intraperitoneally to prevent infection after completing the surgery.

### Next-generation sequencing and sample extraction

Total lung tissue RNA from the nephrectomy mouse models was extracted using Trizol-Reagent (Invitrogen, USA) according to the manufacturer’s instructions. Purified RNA was quantified with an ND-1000 spectrophotometer (Nanodrop Technology, USA) at OD260 nm and qualitied using a Bioanalyzer 2100 (Agilent Technology, USA) with an RNA 6000 LabChip kit (Agilent Technology, USA). The mRNA expression profiles were examined via NGS and performed by AllBiolife Biotech Inc. (Taichung, Taiwan). All RNA samples were prepared according to the official protocol of Illumina, Inc. For the mRNA sequencing, the Agilent SureSelect Strand-Specific RNA Library Preparation Kit was used to build the libraries, and AMPure XP beads (Beckman Coulter, USA) were used to select the sizes. The sequence was read using Illumina’s sequencing-by-synthesis (SBS) technology (Illumina, USA). Sequencing data (FASTQ readings) were generated using a pipeline from Welgene Biotech based on Illumina base call bcl2fastq v2.20. Sequence quality trimming was performed in Trimmomatic version 0.36 to eliminate low-quality read/base data. HISAT2 is a fast and sensitive alignment program used to map next-generation sequencing readings to the entire genome [16].

### RNA sequencing methods

In this study, we conducted gene expression analysis using RNAseq technology. Our methodology began with Quality Control, where raw data in fastq format was preprocessed using Trimmomatic (v0.30) to remove adapters, PCR primers, and low-quality bases (quality score < 20), resulting in clean, high-quality data. For the mapping step, we acquired reference genome sequences and gene model annotation files from genome databases such as UCSC, NCBI, and ENSEMBL, and indexed the reference genome using Hisat2 (v2.0.1). This was followed by aligning the clean data to the reference genome using the same software. Gene expression levels were quantified from the RNAseq raw data using FeatureCounts software. Differential gene expression was analyzed with EdgeR software, identifying genes as differentially expressed based on a False Discovery Rate (FDR) < 0.05 and an absolute Log Fold Change (logFC) > 1. The results were visualized in a volcano plot generated in the R programming language using packages such as EnhancedVolcano. Additionally, we performed functional enrichment analysis of the differentially expressed genes using the Gene Ontology (GO) and Kyoto Encyclopedia of Genes and Genomes (KEGG) databases, providing insights into the biological functions and pathways associated with these genes.

### Differential expression gene (DEG) analysis and pathway enrichment analysis

Differential expression analysis was performed using Cuffdiff (Cufflinks v2.2.1) with genome bias detection/correction [17] and AllBiolife Biotech Inc.’s internal pipeline. The functional enrichment assays for the differentially expressed genes from each experimental design were performed using clusterProfiler v3.6 [18]. Changes in gene expression that were more than doubled and had a p-value of less than 0.05 were considered significantly differentially expressed (DEG). Overrepresentation analyses using Gene Ontology (GO) and the Kyoto Encyclopedia of Genes and Genomes (KEGG) were performed with the compareCluster function in the R package clusterProfiler. Data were normalized using the R package clusterProfiler. A Venn diagram showing the overlap of the genes between the four data sets based on size was generated by Venny 2.1 (https://bioinfogp.cnb.csic.es/tools/venny/index.html) [19].

## Results

### Overrepresentation analysis revealed exercise-induced genetic types by using Gene Ontology

**Fig 1:**
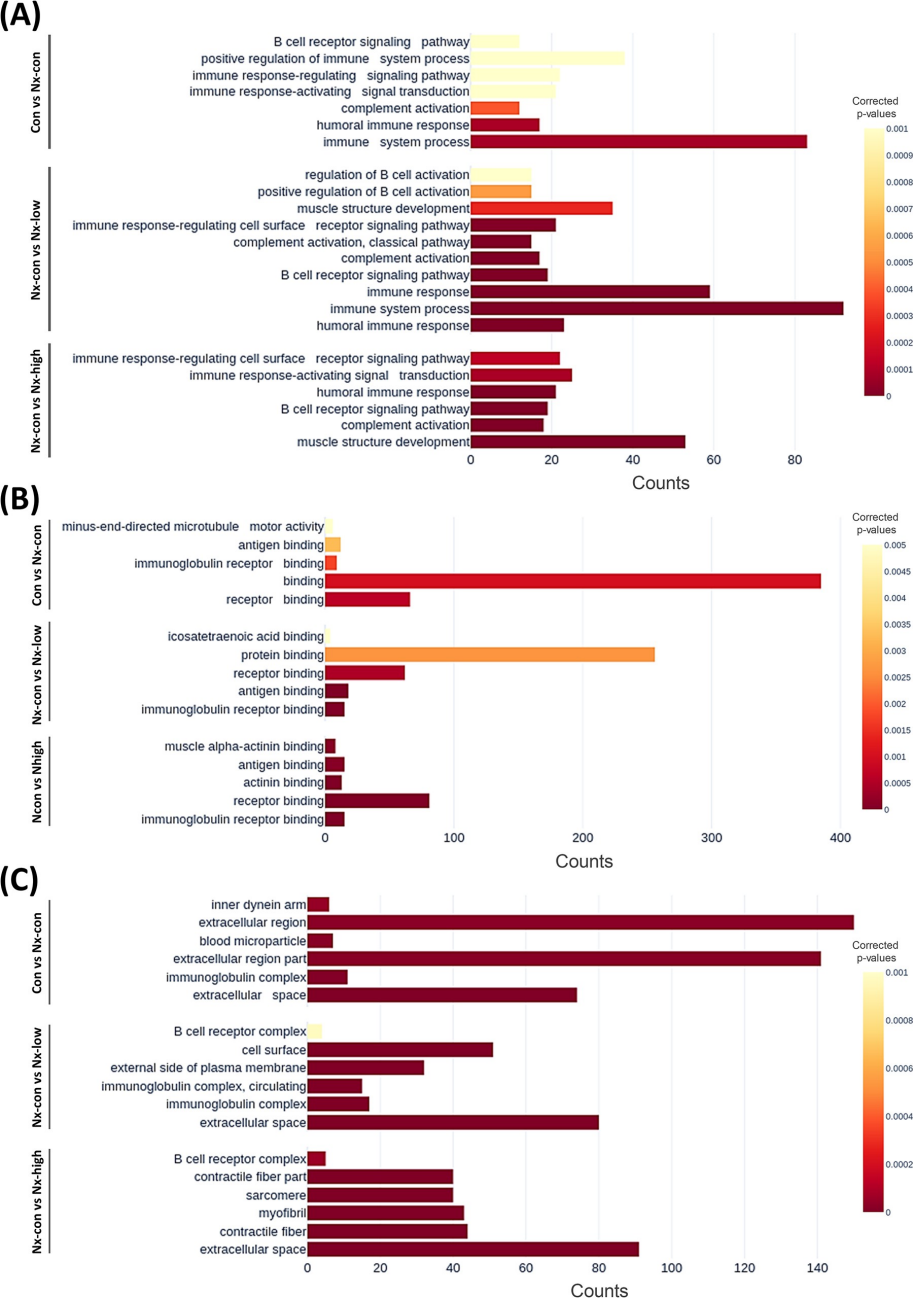
The overrepresentation analysis of pathway enrichment is based on the Gene Ontology database and is represented by three panels: (A) biological processes, (B) molecular functions, and (C) cellular components. Within each panel, the comparison is presented in descending order with three comparison sets: Con vs. Nx, Nx-con vs. Nx-low, and Nx-con vs. Nx-high.

To determine the characteristics of the exercise-induced gene set in our model, we first evaluated the significant gene changes among the Con, Nx-con, Nx-low, and Nx-high groups. We then categorized these genes into different gene sets using the GO term system. As shown in Fig 1, nephrectomy resulted in gene changes related to immunoglobulin receptor binding (molecular function), immunoglobulin complex (cellular component), humoral immune response, immune response-activating signal transduction, and the B cell receptor signaling pathway (biological processes). Differences in gene enrichment between the Nx-con group and the Nx-low/Nx-high groups were found for antigen binding (molecular function), contractile fiber, immunoglobulin complex (cellular components), muscle structure development, complement activation, and the B cell receptor signaling pathway (biological processes).

### Overrepresentation analysis uncovered the impact of exercise on genes by using the Kyoto Encyclopedia of Genes and Genomes

**Fig 2:**
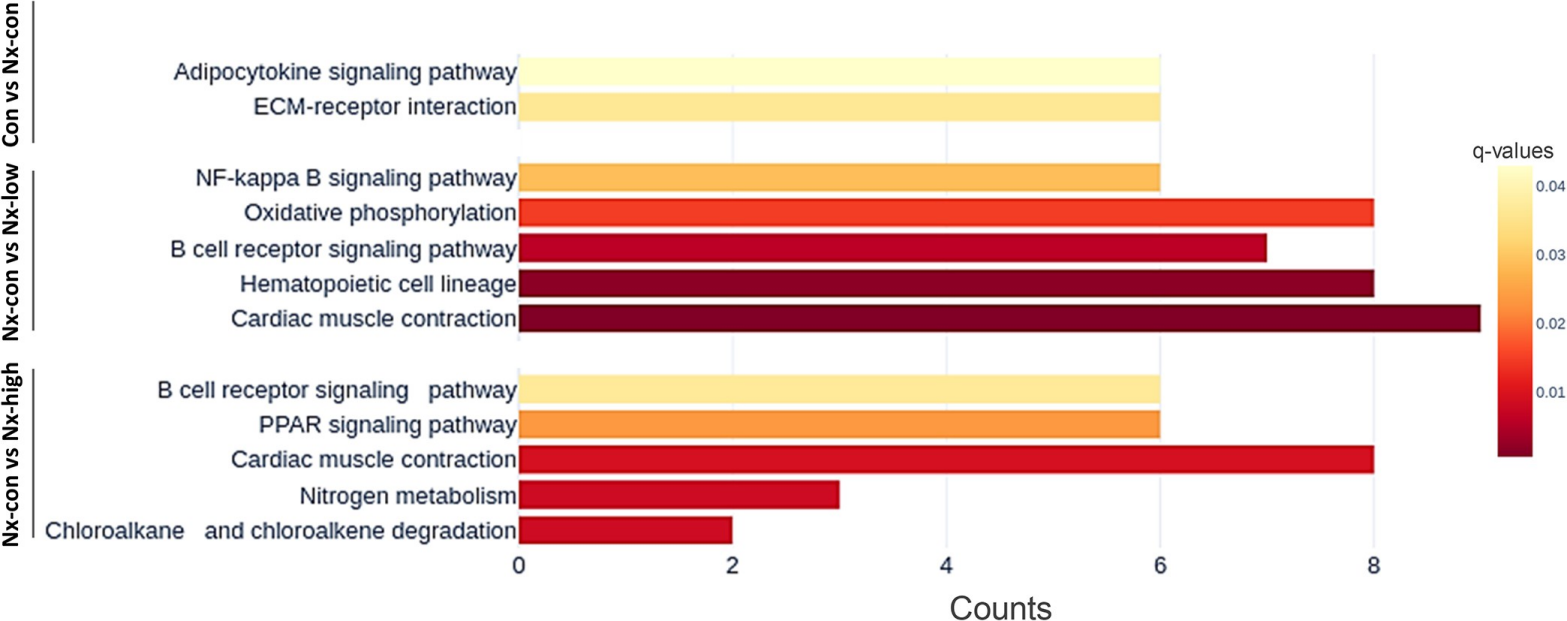
An overrepresentation analysis of path enhancement is based on the Kyoto Encyclopedia of Genes and Genomes database and contains three comparison sets (from upper to lower: Con vs Nx, Nx-con vs Nx-low, Nx-con vs Nx-high).

Next, we utilized the KEGG database to analyze the gene enrichment in our model. The results of the overrepresentation analysis revealed the exercise-involved genes in the post-nephrectomy mouse model (Fig 2 and S2 Fig). Nephrectomy resulted in enrichment related to the adipocyte signaling pathway and the interaction of the extracellular matrix receptor. The exercise-induced enriched pathways included the hematopoietic cell lineage, nuclear factor-kappa-light-chain-enhancer of activated B cells (NF-κB) signaling pathway, cardiac muscle contraction, and peroxisome proliferator-activated receptor (PPAR) signaling pathway.

### Differential expression of nephrectomy-related and exercise-induced genes

**Fig 3:**
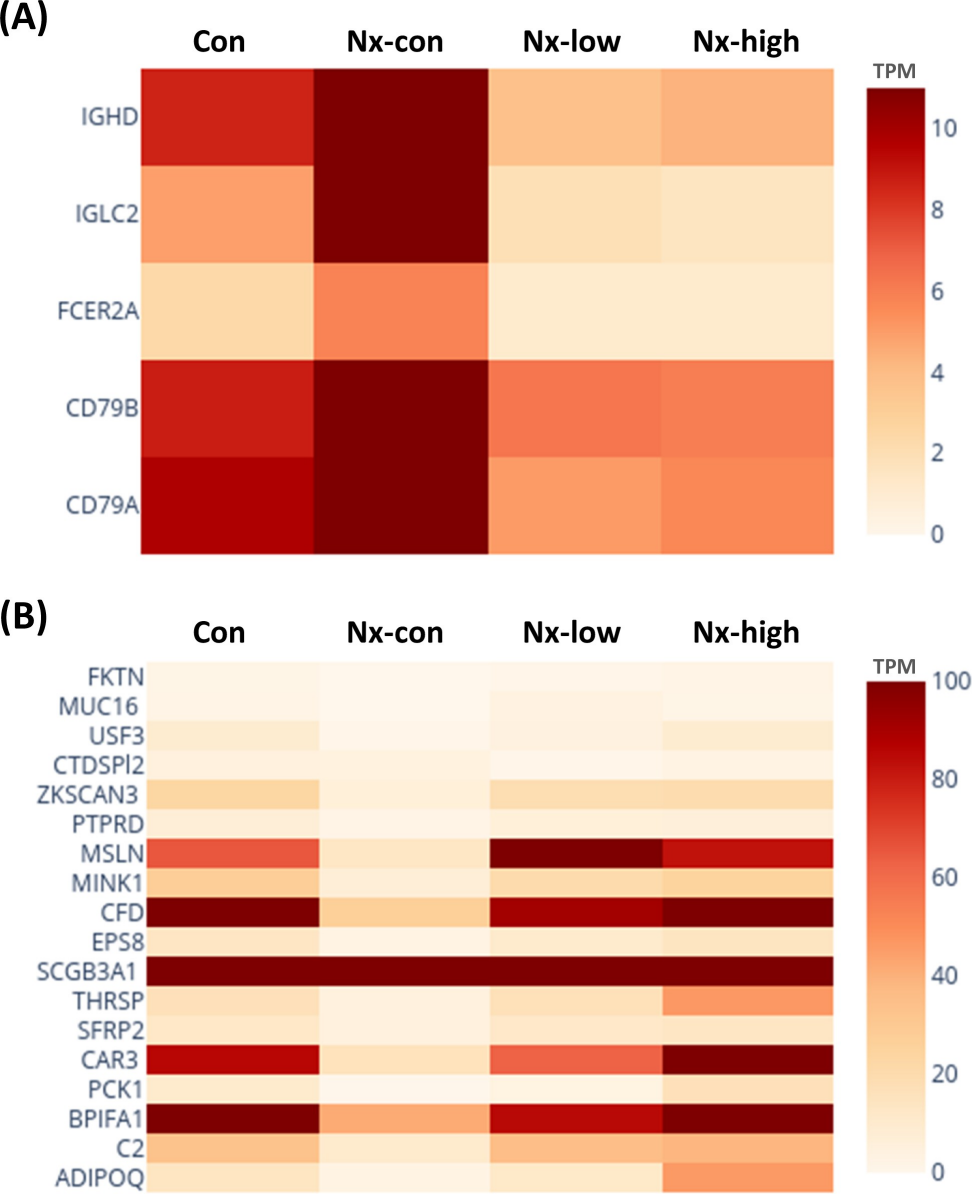
Differential expression of immune-related and cancer-associated genes observed in the intervention groups. The genes shown in (A) indicate upregulation of gene expression in the Nx-con group, which was reduced in the exercise groups. The genes displayed in (B) indicate downregulation of gene expression in the Nx-con group, which increased in the exercise groups. Gene expression levels were quantified using transcripts per million (TPM), with the color intensity reflecting the expression level.

**Fig 4:**
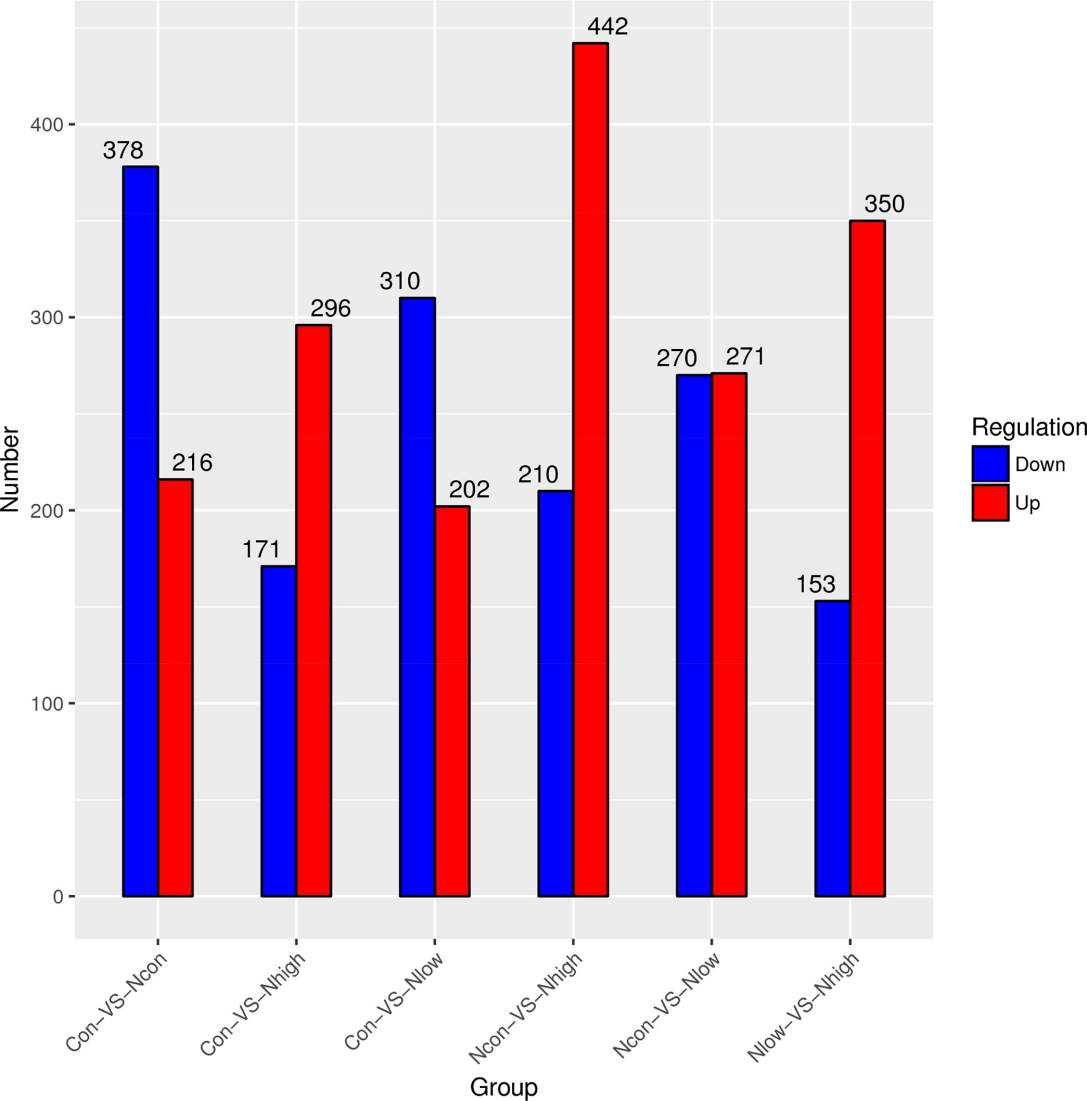
Significant upregulated and downregulated gene numbers are shown. Blue and red represent downregulated and upregulated gene numbers, respectively.

**Fig 5:**
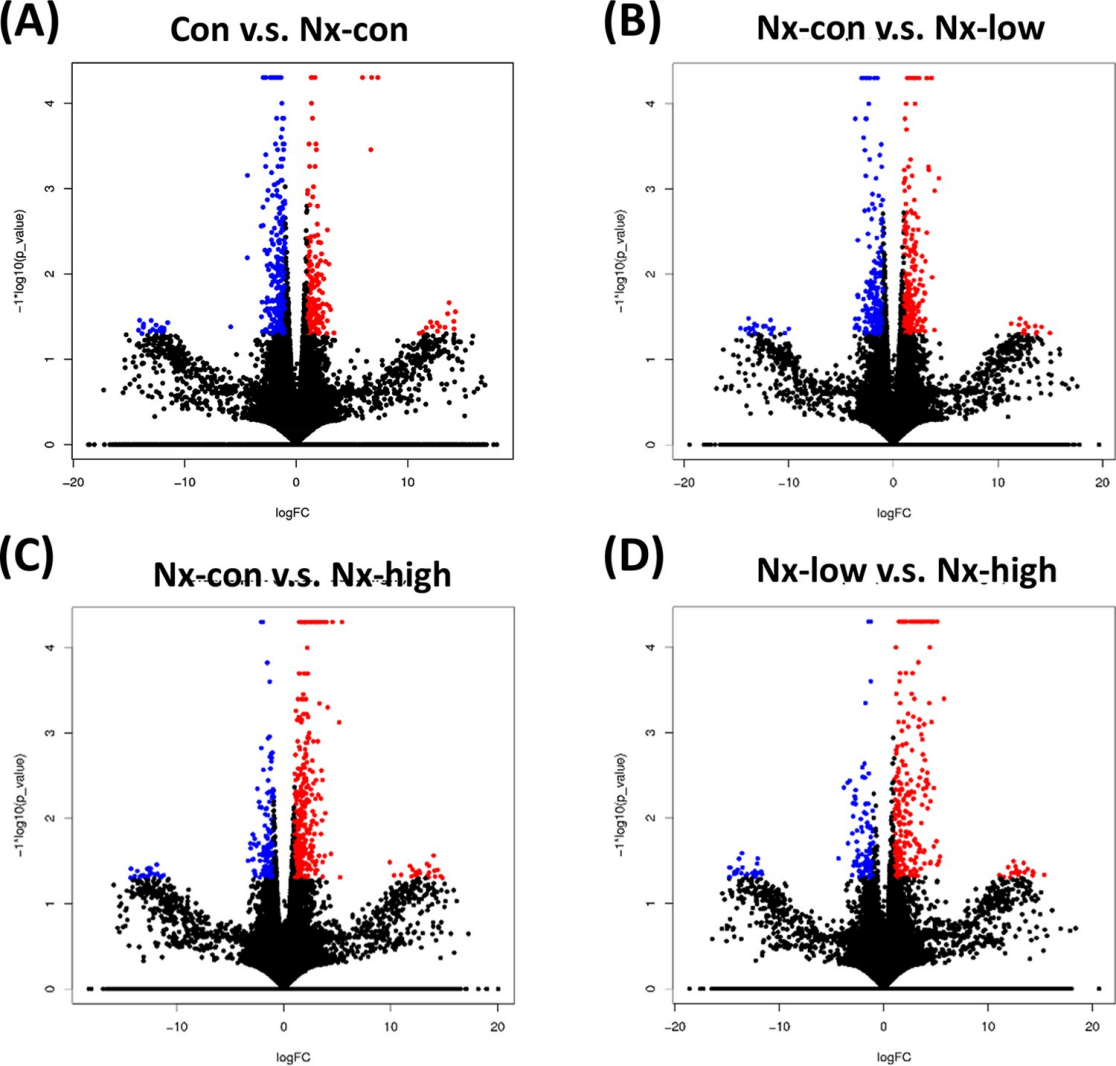
Volcano plot illustrating the fold change in all genes. Red represents significant upregulated genes, and blue represents significant downregulated genes.

To investigate the gene expression changes induced by nephrectomy and exercise, we performed a differential expression analysis of the genes. The heatmap in Fig 3 displays the expression levels of the immune- and cancer-related genes, which are also detailed in Table 1. Heatmap panel (A) demonstrates the upregulation of CD79A and IGHD following nephrectomy, which was subsequently reversed by the exercise intervention. Conversely, the heatmap panel (B) illustrates the suppression of gene expression, such as for EPS8, PCK1, and MINK1, in response to nephrectomy, and their expression was subsequently reversed by the exercise intervention. The number of upregulated and downregulated genes that was statistically significant is shown in Fig 4. The volcano plot in Fig 5 and S3 Fig provides a comprehensive view of the overall changes in gene expression across the different groups.

**Table 1:** Selected immune-related and cancer pathway-related genes in the control group (Con) versus the nephrectomy without exercise group (Nx-con).

Gene	log_2_FC	*p*-value	FDR	Gene function
*Upregulated gene*				
CD79A^**✝**^	3.37	0.0009	0.0553	B cell differentiation and B cell proliferation
CD79B	3.94	0.0001	0.0152	Ig-beta protein of the B-cell antigen component
FCER2A	4.05	0.0002	0.0208	Humoral immune response
IGLC2	5.29	0.00007	0.0089	Activation of immune response
IGHD^**✝**^	2.68	0.011	0.2733	Positive regulation of B cell proliferation
*Downregulated gene*				
ADIPOQ	-6.97	0.00000001	0.000003	negative regulation of gluconeogenesis; and positive regulation of glucose import
C2	-4.11	0.00002340	0.0036	classical pathway of the complement system
BPIFA1	-5.74	0.00000000	0.000002	antibacterial humoral response; innate immune response
SFRP2	-3.62	0.00061221	0.0419	Wnt-protein binding activity
PLCB4	-0.74	0.43040360	0.9975	mitogen-activated protein kinase binding activity
THRSP	-4.73	0.00001972	0.0032	Acts upstream of or within response to bacterium
SCGB3A1	-4.48	0.00000238	0.0005	small secreted protein that may function in the inhibition of cell growth
EPS8^**☨**^	-1.85	0.05182801	0.6061	cellular response to leukemia inhibitory factor
CFD	-6.15	0.00000000	0.00000019	important role in the alternative pathway of complement activation
JAK2	-0.49	0.59696200	0.9975	interleukin-12 receptor binding activity and protein tyrosine kinase activity
MINK1	-1.02	0.27242110	0.9975	Acts upstream of or within negative T cell selection
PTPRD ^**☨**^	-1.55	0.09937305	0.7903	Acts upstream of or within negative regulation of JAK-STAT pathway and regulation of immune response
ZKSCAN3	-1.63	0.08214145	0.7389	Acts upstream of or within autophagy
CTDSPl2	-0.31	0.74614960	0.9975	Acts upstream of or within negative regulation of BMP signaling pathway
USF3 ^**☨**^	-0.037	0.96797050	0.9975	negative regulation of epithelial to mesenchymal transition
MAP1lC3B	0.826	0.37293930	0.9975	Acts upstream of or within autophagy
MUC16	-6.79	0.00000074	0.0002	negative regulation of epithelial cell proliferation, interleukin-6 production, and wound healing
FKTN	-2.71	0.00679559	0.2028	negative regulation of cell population proliferation

✝ Prognostic factor (unfavorable) in renal cancer (human)

☨ Prognostic factor (favorable) in renal cancer (human)

*Prognostic from the human protein atlas (https://www.proteinatlas.org/)

Gene function assessed from National Library of Medicine gene database (*Mus musculus*) (https://www.ncbi.nlm.nih.gov/gene/?term=)

### Reverse gene expression in nephrectomy and exercise shown in an overlapping gene analysis

**Fig 6:**
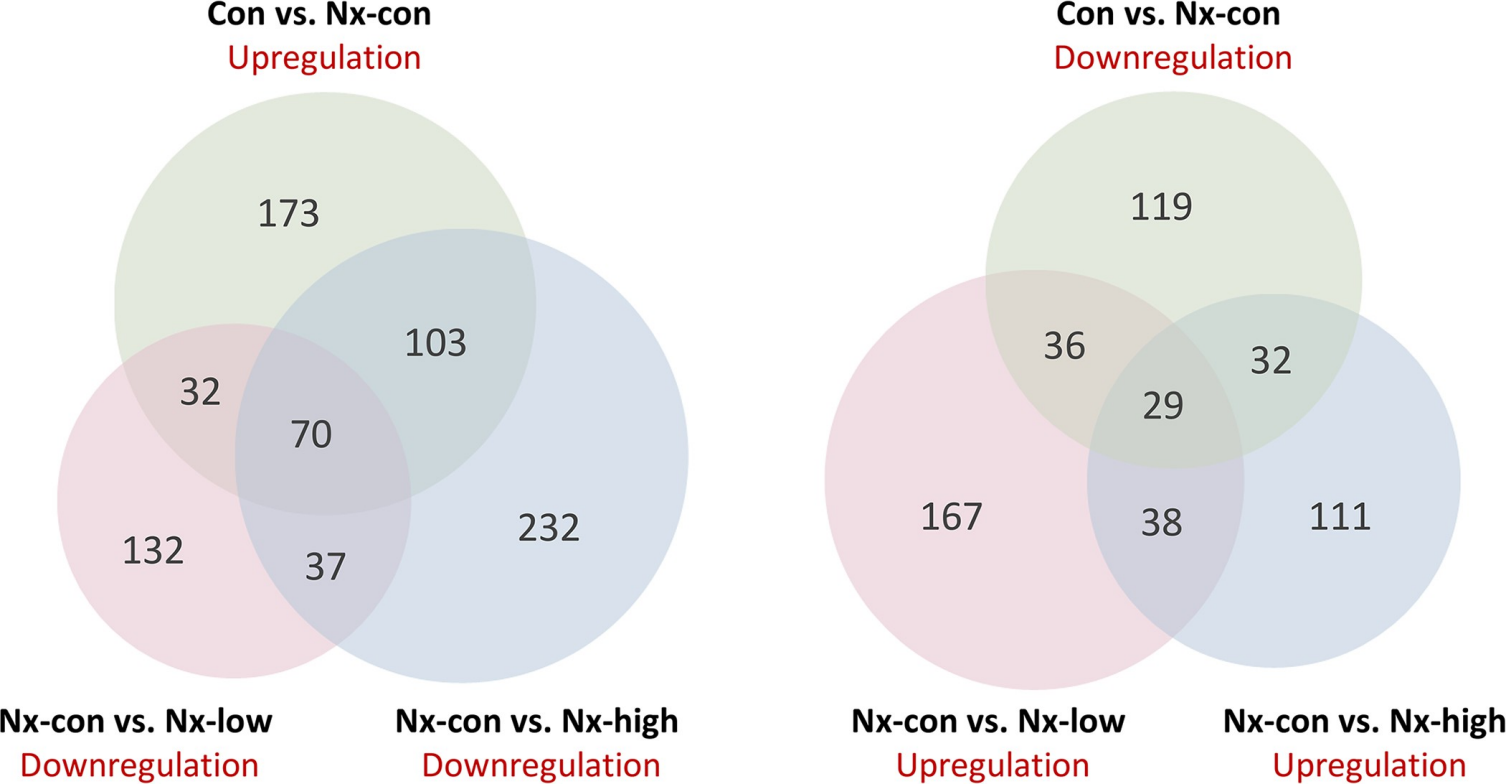
Venn diagram illustrating gene overlapping of up/downregulation of genes in response to nephrectomy and down/upregulation genes in response to exercise.

We hypothesized that exercise could reverse gene changes caused by nephrectomy. Therefore, we analyzed the upregulation of genes caused by exercise (Nx-con group versus Nx-low and Nx-high groups) and the downregulation of genes caused by nephrectomy. We then overlapped three groups of genes that were upregulated and downregulated and identified 70 genes that were upregulated by nephrectomy and downregulated by exercise as well as 29 genes that were downregulated by nephrectomy and upregulated by exercise (Fig 6). Notably, we observed immune-related and cancer-associated pathways among these overlapping genes (Con versus Nx-con) (Table 1). B cell-associated and humoral response genes were upregulated (CD79A, CD79B, FCER2A, and IGHD). T cell-related genes and some cancer inhibition pathway genes, including misshapen-like kinase 1 (MINK1), phosphoenolpyruvate carboxykinase 1 (PCK1), secretoglobin family 3 A member 1 (SCGB3A1), and epidermal growth factor receptor kinase substrate 8 (EPS8), were negatively regulated. We further compared the expression fold change and p-value of these genes between the Nx-con and Nx-high/low groups (Table 2).

**Table 2:** False discovery rate and expression fold change of reverse-regulated genes nephrectomy mice with non-exercise versus exercise group (Nx-con versus Nx-low or high).

	Nx-con versus Nx-low		Nx-con versus Nx-high	
	log_2_FC	FDR	log_2_FC	FDR
*Downregulated gene*				
CD79A^**✝**^	-2	0.00115	-1.91	0.0027
CD79B	-1.55	0.0074	-1.67	0.0078
FCER2A	-2.28	0.00475	-2.31	0.00645
Ighv5-4	-∞	0.02045	-∞	0.0236
Ighv2-5	-∞	0.0362	-∞	0.0391
Ighv3-1	-∞	0.01145	-∞	0.01345
IGLC2	-2.53	0.01235	-2.89	0.01545
IGHD^**✝**^	-2.26	0.00045	-2.09	0.0015
Igkv9-124	-∞	0.00315	-∞	0.0042
*Upregulated gene*				
ADIPOQ	2.09	0.0047	3.97	0.00005
C2	1.78	0.0019	1.81	0.0054
BPIFA1	1.07	0.0021	5.44	0.00005
PCK1^**☨**^	3.06	0.00705	5.18	0.00075
CAR3	2.02	0.00005	3.9	0.00005
SFRP2	1.23	0.01685	1.31	0.0212
PLCB4	13.57	0.0407	14.4	0.0402
THRSP	1.79	0.00345	3.19	0.00005
SCGB3A1	1.08	0.0008	3.21	0.00005
EPS8^**☨**^	1.85	0.0072	2.16	0.006
CFD	1.8	0.0007	3.37	0.00005
JAK2	1.4	0.0028	1.23	0.0171
MINK1	1.55	0.04965	1.73	0.0479
MSLN ^**☨**^	3.19	0.00005	2.6	0.00005
Ighv11-2	∞	0.01645	∞	0.0237
PTPRD ^**☨**^	1.54	0.01705	1.53	0.03125
ZKSCAN3	1.63	0.0052	12.04	0.0398
CTDSPL2	12.01	0.03925	12.01	0.03925
USF3 ^**☨**^	2.31	0.0151	2.31	0.0151
MAP1lC3B	∞	0.0331	∞	0.0331
MUC16	3.94	0.00105	3.94	0.00105
FKTN	12.9	0.0416	12.9	0.0416

## Discussion

Exercise has emerged as a crucial component of cancer rehabilitation [11]. The potential benefits of exercise after cancer surgery have been evaluated in several studies [20, 21]. In our study, our objective was to determine the impact of exercise in a post-nephrectomy patient with renal cell carcinoma. Therefore, we first developed an animal model that mimicked post-nephrectomy conditions. Next, we intervened with exercise of different intensities and then extracted lung tissue mRNA to perform next-generation sequencing to examine the genetic differences. To our knowledge, our study seems to be the first bioinformatic analysis to show the genetic impact of exercise on a post-nephrectomy animal model.

The impact of exercise as an intervention has been reported in several studies, including for cancer prevention, immune system regulation, and diseases such as Parkinson’s disease and pulmonary fibrosis [22–24]. In a previous study, the underlying mechanisms of exercise that affect cancer consisted of immune system modulation, energy metabolism, the inhibition of cancer cell proliferation, and the inducement of tumor cell apoptosis [4, 12, 25, 26]. In a breast cancer animal model, exercise improved muscle weakness and reversed mitochondrial gene expression, suggesting that an exercise intervention may help cancer patients improve muscle weakness and decrease the impact of cachexia [27]. Our analysis showed pathway enrichment related to muscle structure development and cardiac muscle contraction in nephrectomy mice after exercise (Figs 1 and 2).

The lungs serve as the primary site for metastatic spread in renal cell carcinoma, affecting nearly half of all patients [28, 29]. The lung microenvironment significantly influences metastatic progression in various cancers, including melanoma [30]. The metastatic process to the lungs is facilitated by several factors, including cancer-derived exosomes, inflammatory monocytes, and fibroblasts [31]. Our objective was to explore changes in the proportion of pulmonary immunity following exercise. Therefore, our research focused on identifying transcriptional changes in the pulmonary immune environment by analyzing lung tissue in our model.

Given that exercise has immune system effects that have been observed in previous studies, we highlight the immune-related pathways [32, 33]. The NF-κB pathway plays an important role in immune function, and its association with exercise has been documented in previous studies [34, 35]. Notably, resistance exercise has been shown to activate NF-κB binding activity, thereby influencing the immune system [36]. Our KEGG analysis revealed the enrichment of the NF-κB signaling pathway in the exercise groups (Fig 2). The B cell-related pathway was enriched in both the nephrectomy and exercise groups (Fig 1).

In our analysis of differential genes, we identified genes that exhibited increased expression in the nephrectomy group but were suppressed in the exercise groups (Fig 5). The expression of CD79A and IGHD, which are responsible for B cell differentiation and B cell proliferation, were upregulated in the nephrectomy mice (Table 2). The influence of exercise on B cells has been observed in a few studies. Vigorous exercise changes the expression of CD39 in B lymphocytes [37]. The B cell receptor signaling pathway is enriched in the placenta tissue of pregnant women after recreational physical activity [38]. CD95^+^ B cells do not show significant changes in acute exercise in the bloodstream of adolescents or adults [39].

Previous studies have revealed an exercise-mediated antitumor effect through the mobilization of T cells in the circulation and the reduction of dysfunctional T cells [8, 40]. In a breast cancer mouse model, exercise increased CD8^+^ T cell infiltration and exerted an antitumor effect through the CXCR3-mediated pathway, driving an improvement in tumor control and sensitivity to immunotherapy [41]. In our study, we observed the negative regulation of PCK1 and MINK1, which are genes associated with T cells, in the nephrectomy-treated groups. However, reversed expression was observed in the exercise groups (Table 1). PCK1 is a protein kinase that mediates energy metabolism and epigenetic modification in CD8^+^ memory T cells [42]. However, a study by Cao *et al*. was inconsistent with our results and did not show a significant change in PCK1 expression level after a high-intensity interval exercise in a hepatocellular carcinoma animal model [39]. Nevertheless, another study demonstrated that the expression level in liver tissue increased after exercise in mice fed a high-fat diet [43]. MINK1 has been shown to be involved in the process of T helper 17 cell differentiation and linked to the oncogenic transformation of human ovarian epithelial cells [44, 45].

In addition to evaluating immune-related pathways, we further investigated tumor-related genes. The expression levels of SCGB3A1, EPS8, and USF3 showed a surge pattern after exercise (Table 2). SCG3B3A1, a tumor suppression secretoglobin, is decreased in squamous cell carcinoma compared to normal lung epithelium in smokers, and the loss of function in this protein is associated with a poor prognosis in lung cancer [43, 46]. Furthermore, SCG3B3A1 showed the ability to inhibit the cell cycle, inducing apoptosis and abating cell migration in a breast cancer cell line [44]. The knockdown of USF3 contributes to tumor cell survival and enhances the epithelial-to-mesenchymal transition, thereby contributing to tumor cell survival. However, contrary to our expectations, EPS8 is a protein involved in cell proliferation and, when inhibited, the sensitivity of the chemotherapeutic agent increases in several tumor cell lines [45, 46].

The strength of our study is restricted by several shortcomings. First, our animal model did not fully replicate the patient who has undergone nephrectomy as we did not implant a tumor in the nephrectomy mice. Furthermore, limiting the study to only male mice reduces the generalizability of the results. Next, we did not provide any further validation of the effects of blood biochemistry study, serum immune profiles, urinalysis, immune functional assay, exercise, including assessing muscle strength or physical activity. Furthermore, the lack of a larger sample size in both the control and experimental groups makes the reproducibility of our results uncertain. External validation is required to verify our findings. Finally, the molecular mechanism of the exercise effect needs further experimentation to confirm its validity.

In summary, our results have highlighted the genetic differences in the immune-related pathways of nephrectomy mice with or without treadmill exercise. The B cell-related and several tumor-suppressing pathways were enhanced in the nephrectomy group after exercise. This study has revealed a piece of the genetic landscape of post-nephrectomy mice with and without an exercise intervention, and this study can provide novel insights and potential mechanisms for future research on exercise and cancer rehabilitation.

## Supporting information

S1 Fig Flow chart depicting our experimental design. (DOCX)

S2 Fig The analysis of pathway enrichment overrepresentation utilizes the Gene Ontology and Kyoto Encyclopedia of Genes and Genomes databases, employing the TPM method. (DOCX)

S3 Fig Volcano plot showcasing fold change across all analyzed genes using False Discovery Rate (FDR) criteria. (DOCX)
