# Supplementary material for: Exercise may improve lung immunity after surgical stress: Evidence from a nephrectomy model via a bioinformatic analysis
Source: PLoS One. 2024 Jun 7;19(6):e0303334. doi: 10.1371/journal.pone.0303334 (PMC11161109; doi:10.1371/journal.pone.0303334)
Supplement: S1 Fig — (DOCX) [file pone.0303334.s001.docx]

**Supporting Information**
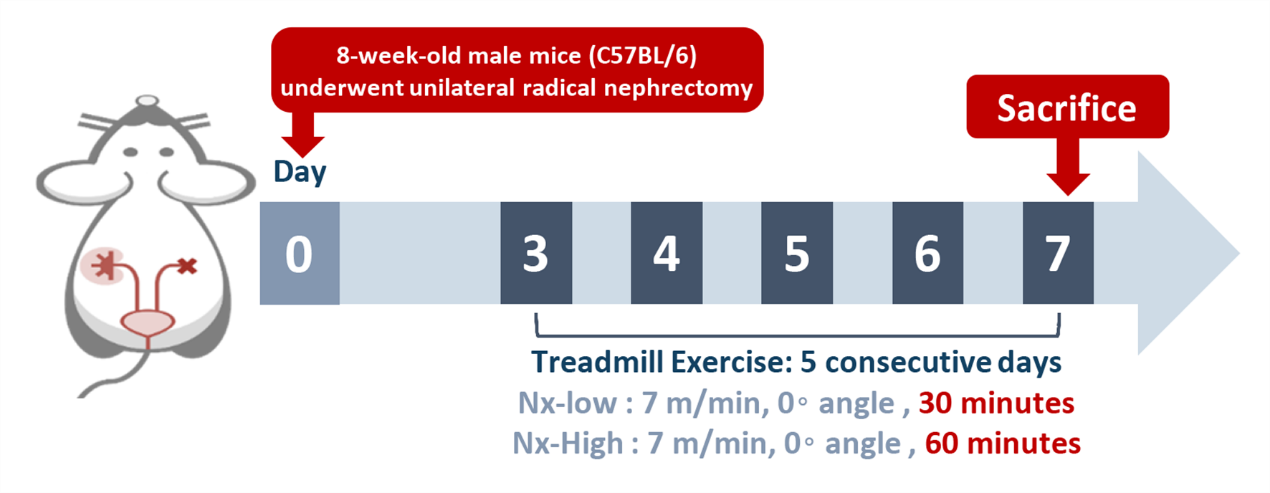


Figure S1. Flow chart depicting our experimental design. Mice were subjected to exercise training on a treadmill at a speed of 7 m per minute at a 0° inclination for either 30 or 60 minutes. A 5-day adaptation period was implemented, during which the mice exercised for 10 minutes each day at a speed of 5 m per minute to minimize stress. Following this adaptation period, a nephrectomy was performed, succeeded by a 3-day recovery period. After recovery, the mice underwent treadmill exercise sessions daily for 5 consecutive days, each session lasting either 30 or 60 minutes. The mice were sacrificed on the fifth day of the experiment following the exercise sessions.
