# Supplementary material for: Exercise may improve lung immunity after surgical stress: Evidence from a nephrectomy model via a bioinformatic analysis
Source: PLoS One. 2024 Jun 7;19(6):e0303334. doi: 10.1371/journal.pone.0303334 (PMC11161109; doi:10.1371/journal.pone.0303334)
Supplement: S2 Fig — (DOCX) [file pone.0303334.s002.docx]

**Supporting Information**


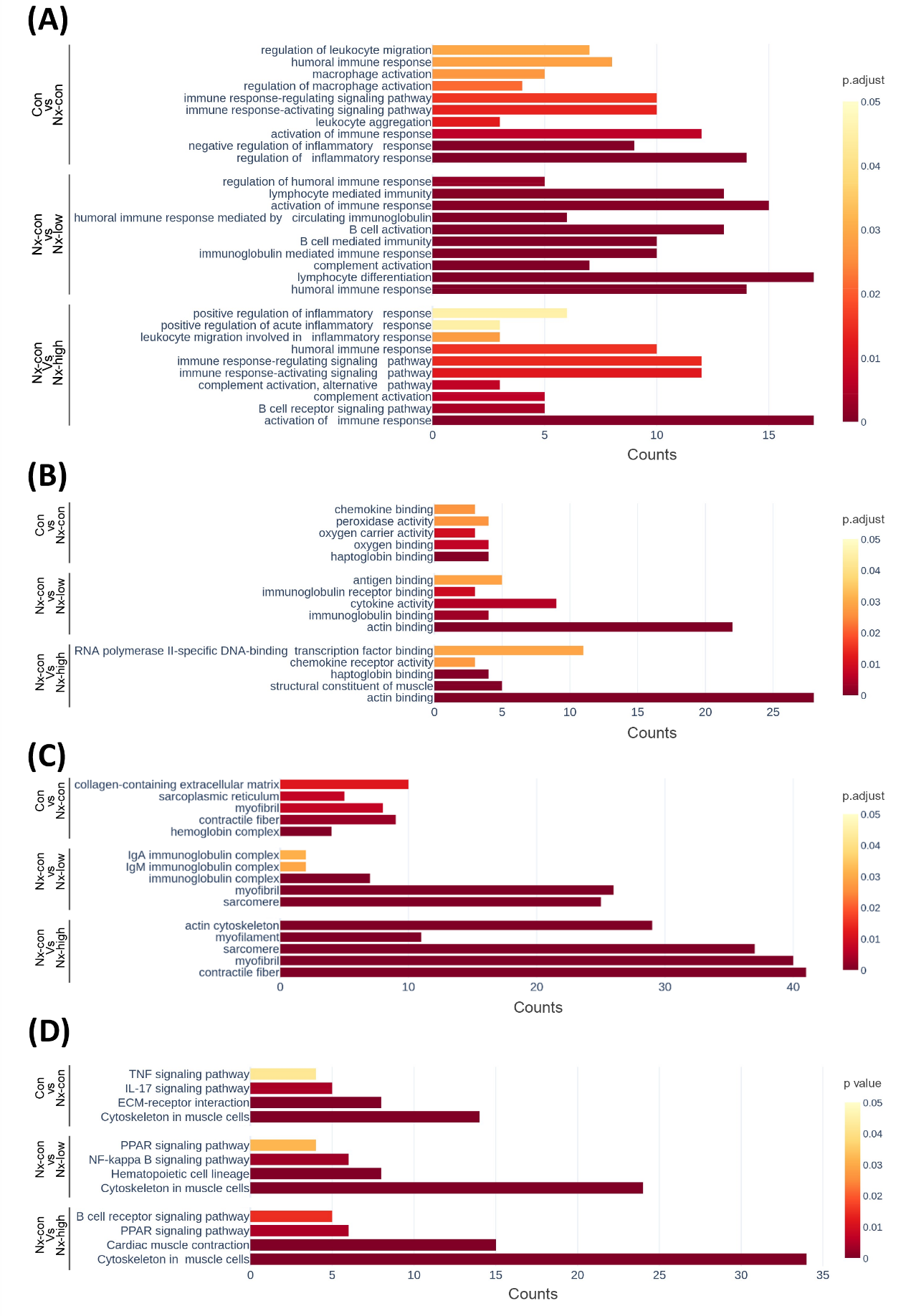


Figure S2. The analysis of pathway enrichment overrepresentation utilizes the Gene Ontology and Kyoto Encyclopedia of Genes and Genomes databases, employing the TPM method. It is depicted across four panels: (A) biological processes, (B) molecular functions, (C) cellular components, and (D) KEGG pathways. Each panel presents the data in descending order for three comparative sets: Con vs. Nx, Nx-con vs. Nx-low, and Nx-con vs. Nx-high.
