# Supplementary material for: Exercise may improve lung immunity after surgical stress: Evidence from a nephrectomy model via a bioinformatic analysis
Source: PLoS One. 2024 Jun 7;19(6):e0303334. doi: 10.1371/journal.pone.0303334 (PMC11161109; doi:10.1371/journal.pone.0303334)
Supplement: S3 Fig — (DOCX) [file pone.0303334.s003.docx]

**Supporting Information**


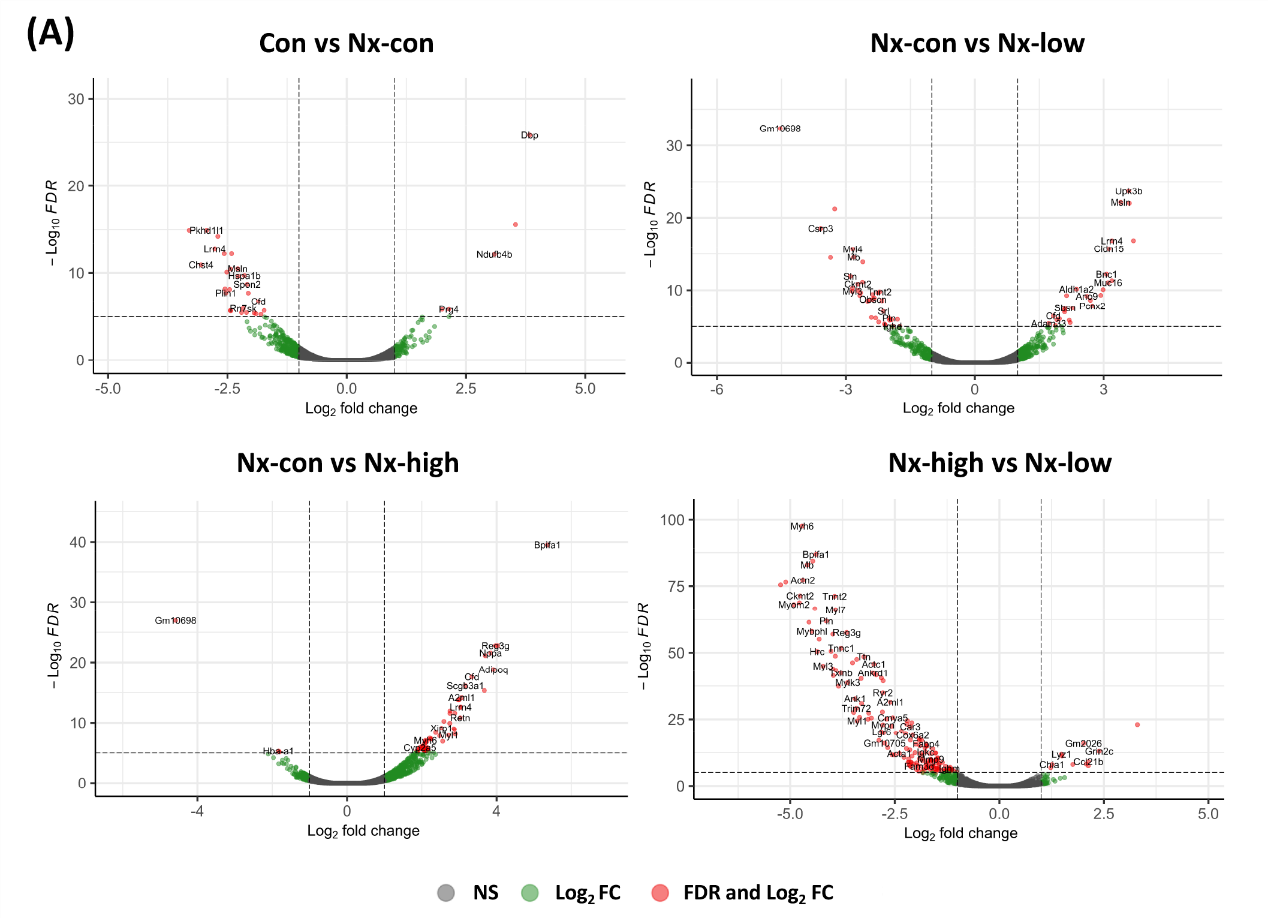


Figure S3. Volcano plot showcasing fold change across all analyzed genes using False Discovery Rate (FDR) criteria. In this visualization, genes that are significantly upregulated or downregulated are highlighted in red. Genes that do not meet the significance threshold are marked in green.
